# Spin memristive magnetic tunnel junctions with CoO-ZnO nano composite barrier

**DOI:** 10.1038/srep03835

**Published:** 2014-01-23

**Authors:** Qiang Li, Ting-Ting Shen, Yan-Ling Cao, Kun Zhang, Shi-Shen Yan, Yu-Feng Tian, Shi-Shou Kang, Ming-Wen Zhao, You-Yong Dai, Yan-Xue Chen, Guo-Lei Liu, Liang-Mo Mei, Xiao-Lin Wang, Peter Grünberg

**Affiliations:** 1 School of Physics, National Key Laboratory of Crystal Materials, Shandong University, Jinan, 250100, P. R. China; 2 Institute of Superconducting and Electrical Materials, Australian Institute of Innovative Materials, University of Wollongong, Wollongong, NSW 2522, Australia; 3 Peter Grünberg Institute, Forschungszentrum Jülich, Wilhelm-Johnen-Straβe, Jülich, 52428, Germany

## Abstract

The spin memristive devices combining memristance and tunneling magnetoresistance have promising applications in multibit nonvolatile data storage and artificial neuronal computing. However, it is a great challenge for simultaneous realization of large memristance and magnetoresistance in one nanoscale junction, because it is very hard to find a proper spacer layer which not only serves as good insulating layer for tunneling magnetoresistance but also easily switches between high and low resistance states under electrical field. Here we firstly propose to use nanon composite barrier layers of CoO-ZnO to fabricate the spin memristive Co/CoO-ZnO/Co magnetic tunnel junctions. The bipolar resistance switching ratio is high up to 90, and the TMR ratio of the high resistance state gets to 8% at room temperature, which leads to three resistance states. The bipolar resistance switching is explained by the metal-insulator transition of CoO_1−v_ layer due to the migration of oxygen ions between CoO_1−v_ and ZnO_1−v_.

Giant magnetoresistance, the giant change of electrical resistance of magnetic multilayer films in magnetic field, has led to the great leap in revolution of the hard drive data storage devices and the birth of spintronics. In order to satisfy the ever-increasing demand for faster, smaller, and non-volatile electronics in information storage and processing technology, various novel electronic devices such as spin field-effect transistors^1^, multiferroic memories^2^, racetrack memories^3^,^4^, and memristors^5^,^6^,^7^ have been proposed and investigated. Especially, the new concept of combining memristance and magnetoresistance^8^,^9^,^10^,^11^,^12^,^13^,^14^,^15^ are attracting increasing attention due to their potential applications in multibit nonvolatile data storage and artificial neuronal computing. By electrical and magnetic controlling, multiple resistance states can be achieved in this system, presenting new possibilities towards enhancing data densities by many folds. However, it is a great challenge for simultaneous realization of large memristance and magnetoresistance in one junction at room temperature, because it is very hard to find a proper spacer layer which not only serves as good insulating layer for tunneling magnetoresistance but also easily switches between a high resistance state and a low resistance state under electrical field. On the first hand, tunneling magnetoresistance (TMR) has been used in read-heads and magnetic random access memory, but the TMR junctions are limited to very few insulating materials such as Al_2_O_3_^16^ and MgO^17^ of 1–3 nm in thickness. On the other hand, transition metal oxides such as NiO^18^,^19^, Ta_2_O_5_^20^, Fe_2_O_3_^21^, and CoO^22^,^23^, have been regarded as promising candidates for memristance materials due to their high resistance ratio between the high and the low resistance states, simple constituents, and compatibility with complementary metal oxide semiconductors, but they are usually not good insulating materials for TMR junctions. Therefore, simultaneous realization of large memristance and magnetoresistance in one junction at room temperature is highly desirable.

Here we proposed a novel system of Co/CoO-ZnO/Co magnetic tunnel junctions (MTJs) with CoO-ZnO nanon composite barrier layers, which can simultaneously show large memristance and magnetoresistance due to the metal-insulator transition of CoO_1−v_ (v denotes O vacancies) layer controlled by the migration of oxygen ions (or O vacancies) between CoO_1−v_ and ZnO_1−v_. The system has two obvious advantages: First, a very thin compact CoO insulating layer is formed as tunneling barrier by depositing a very thin ZnO layer onto the bottom Co layer, and the ZnO layer acts as a reservoir of O ions (or O vacancies) for the electrical switching of CoO under applied electrical field. Second, the insulating antiferromagnetic CoO layer can supply an exchange bias field on its bottom Co layer, which can be used to control the magnetization reversal of the bottom Co layer to realize the spin-dependent function of the devices, such as tunneling magnetoresistance. In this communication, three resistance states are obtained in Co/CoO-ZnO/Co magnetic tunnel junctions by combining memristance and tunneling magnetoresistance. The bipolar resistance switching ratio of the high resistance to the low resistance is high up to 90 at room temperature. Moreover, the junctions in the high resistance state simultaneously show a tunneling magnetoresistance ratio of 8% at room temperature.

## Results

**Figure 1:**
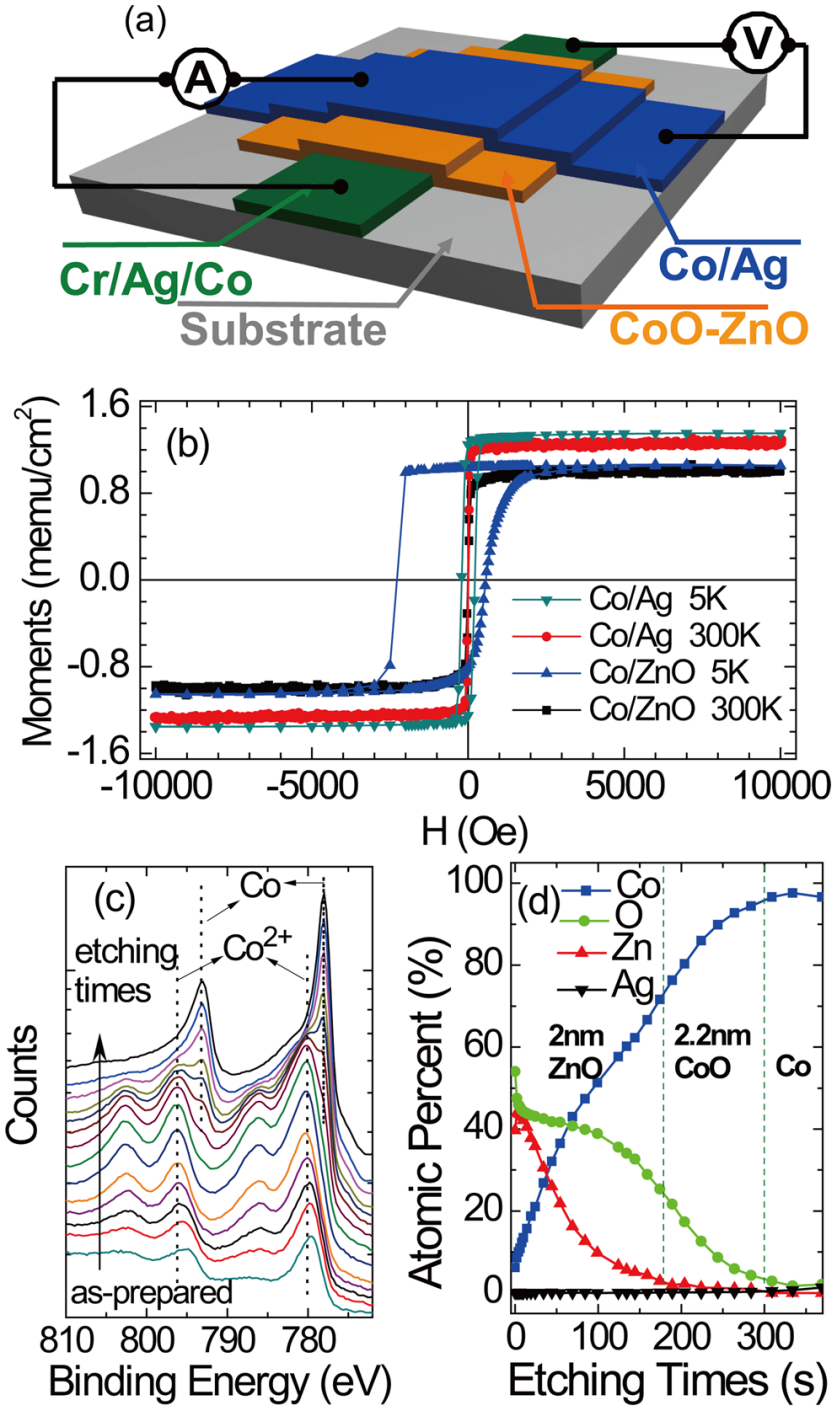
(a) Schematics of the junction structure and measuring configuration. (b) Hysteresis loops of the glass/Cr(2 nm)/Ag(30 nm)/Co(10 nm)/CoO-ZnO(2 nm)/Ag(60 nm) film (marked as Co/ZnO) and the glass/Cr(2 nm)/Ag(30 nm)/Co(10 nm)/Ag(60 nm) reference film (marked as Co/Ag) measured by SQUID at 5 K and 300 K. The Hysteresis loops at 5 K were measured after cooling down from 300 K with 30000 Oe magnetic field. (c) The XPS of Co element, which was measured when the glass/Cr(2 nm)/Ag(30 nm)/Co(10 nm)/CoO-ZnO(2 nm) film was etched gradually from the ZnO layer through CoO to Co layer. (d) The atomic percent of Co, O, Zn, and Ag elements measured by XPS, which gradually varies with etching times. In the CoO-ZnO composite layers, Ag is ignorable within the errors of experiments.

The Co/CoO-ZnO/Co MTJs with an area of 0.1 mm × 0.1 mm were depicted schematically in Figure 1a. The structure of junction is glass/Cr(2 nm)/Ag(30 nm)/Co(10 nm)/CoO-ZnO(2 nm)/Co(30 nm)/Ag(60 nm). The top 30 nm Co layer has a larger coercivity (H_C_ = 310 Oe) at room temperature than that of the bottom 10 nm Co layer (H_C_ = 70 Oe), which enables the magnetization of the two Co layers parallel or antiparallel under external magnetic field. An antiferromagnetic insulating CoO thin layer was produced on the surface of the bottom Co layer during the deposition of ZnO, which is similar to the oxidation of Co electrode in magnetic tunnel junctions with ZnO barrier^24^.

Figure 1b shows the hysteresis loops of the glass/Cr/Ag/Co/CoO-ZnO/Ag film and the glass/Cr/Ag/Co/Ag reference film measured by SQUID at 5 K and 300 K, respectively. The saturation magnetization only increases about 3–4% for both films as the temperature decreases from 300 K to 5 K. After cooling down from 300 K to 5 K at the magnetic field of 30000 Oe, a large exchange bias of 850 Oe in Co/CoO-ZnO/Ag film was observed due to the formation of the antiferromagnetic CoO, while no exchange bias was observed in the Co/Ag reference film. The saturation magnetization per unit area of the Co/CoO-ZnO/Ag film is 22% smaller than that of the reference Co/Ag film, which means that 2.2 nm Co layer forms CoO antiferromagnetic insulating layer.

The formation of CoO was further confirmed by X-ray photoelectron spectroscopy (XPS) measurements, as shown in Figure 1c. The XPS of Co element was measured when the Co(10 nm)/CoO-ZnO(2 nm) film was etched gradually from the ZnO layer through CoO to Co layer. After the ZnO layer was etched, the peaks of Co^2+^2p3/2 locating at 780.2 eV, Co^2+^2p1/2 locating at 796.2 eV, and the corresponding satellite peaks confirmed the formation of CoO. After the CoO was etched, we only observed the peaks of Co pure metal Co2p3/2 locating at 778.0 eV and Co2p1/2 locating at 793.1 eV without satellite peaks.

Figure 1d shows the atomic percent of Co, O, Zn, and Ag elements measured by XPS, which gradually varies with etching times. It is clear that the atomic percent of Zn gradually reduces and almost disappears until the ZnO layer was completely etched. The atomic percent of O also reduces and almost disappears until the CoO layer was completely etched. Correspondingly, the atomic percent of Co always increases and approaches saturation until only the Co pure metal layer was left. However, in the CoO-ZnO composite layers, Ag is ignorable within the errors of experiments.

**Figure 2:**
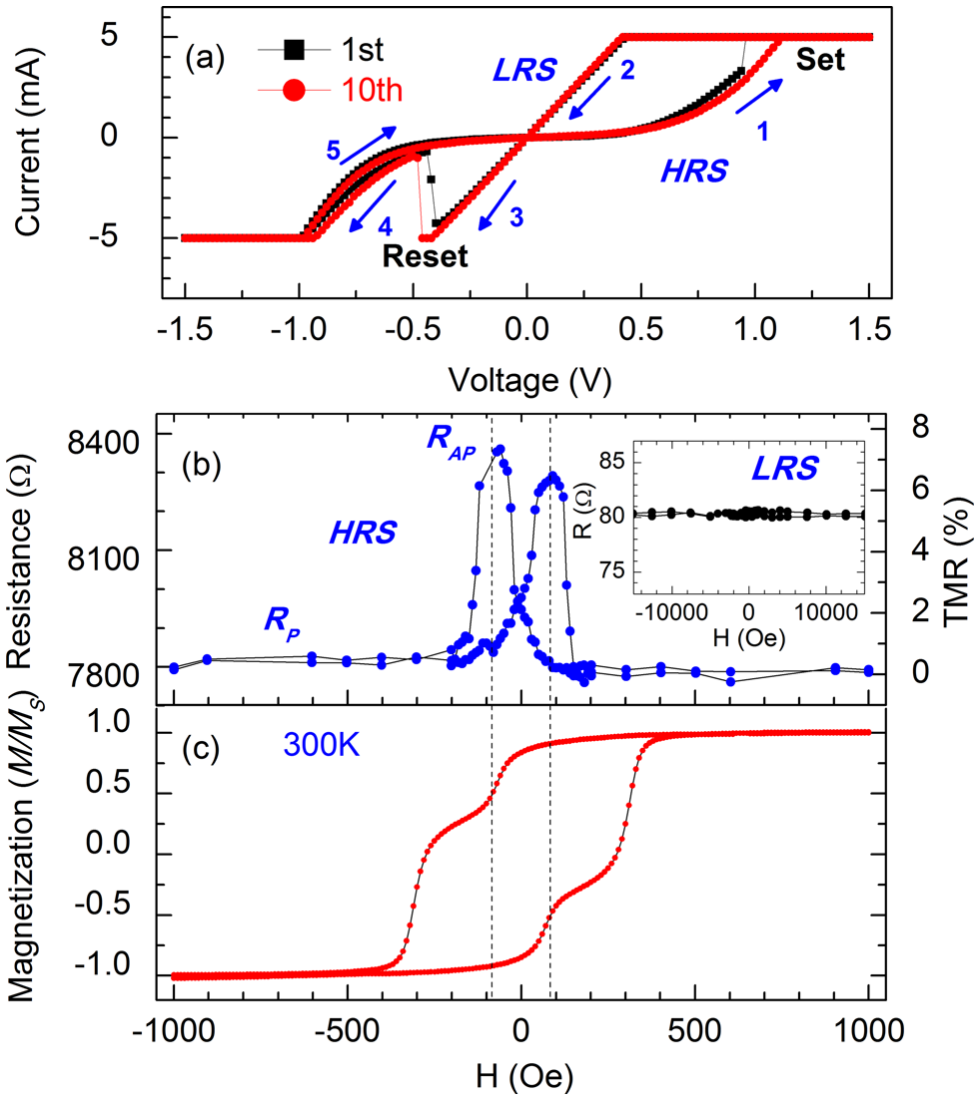
(a) The I-V characteristic of Ag(30 nm)/Co(10 nm)/CoO-ZnO(2 nm)/Co(30 nm)/Ag(60 nm) junction with area 0.1 mm × 0.1 mm, and (b) the tunneling magnetoresistance of the junction. The inset in (b) shows the R-H curve of the low resistance state. (c) The magnetic hysteresis loop of the same junction with larger area of 5 mm × 5 mm. All the data were measured at 300 K.

Figure 2a displays the current-voltage (I-V) characteristic of the junction at room temperature. A current compliance of 5 mA is applied to avoid dielectric breakdown of the device. The voltage was scanned along 1-2-3-4-5 as denoted in Figure 2a. The I-V curve was initially nonlinear in the as-prepared junction, which indicates the tunneling transport. A bipolar resistive switching was observed without any electroforming process in advance. Obviously the positive bias voltage (Set voltage), which was defined by the current flowing from the top electrode to the bottom electrode of the junction, switched the MTJ into a low resistance state (LRS), while the negative bias voltage (Reset voltage) tuned the MTJ into a high resistance state (HRS) abruptly. The resistance ratio of the HRS to LRS is about 90 around zero voltage at room temperature, and the resistive switching repeatability is demonstrated by the comparison between the 1st circle and the 10th circle.

In addition to the two states of HRS and LRS, the junction resistance can also be modulated by magnetic field. The tunneling magnetoresistance loop of high resistance state at room temperature is shown in Figure 2b. The peak tunneling magnetoresistance ratio is about 8%, which is defined as TMR = (R*_AP_* − R*_P_*)/R*_P_*, where R*_AP_* and R*_P_* are the resistance of the junction corresponding to the antiparallel and parallel states of magnetization in the two Co layers. Comparing Figure 2b with Figure 2c indicates that the sharp peaks of resistance around ±70 Oe is due to the magnetization reversal of the bottom Co layer with relatively small coercivity. It is worthy mention that the measuring temperature of 300 K is above the Néel temperature 293 K of bulk material CoO^25^ and no exchange bias was found in the magnetization hysteresis loop, as shown in Figure 2c.

However, there is no magnetoresistance in the LRS as shown in the inset of Figure 2b, and a TMR always appears after switching to the HRS. Thus, Figures 2a and 2b indicate that three resistance states can be obtained by electrical and magnetic controlling: depending on the history of the applied electrical field, the Co/CoO-ZnO/Co MTJ can show the HRS and LRS; depending on the history of the applied magnetic field, the HRS can further involve into HRS-R*_AP_* and HRS-R*_P_* two resistance states.

**Figure 3:**
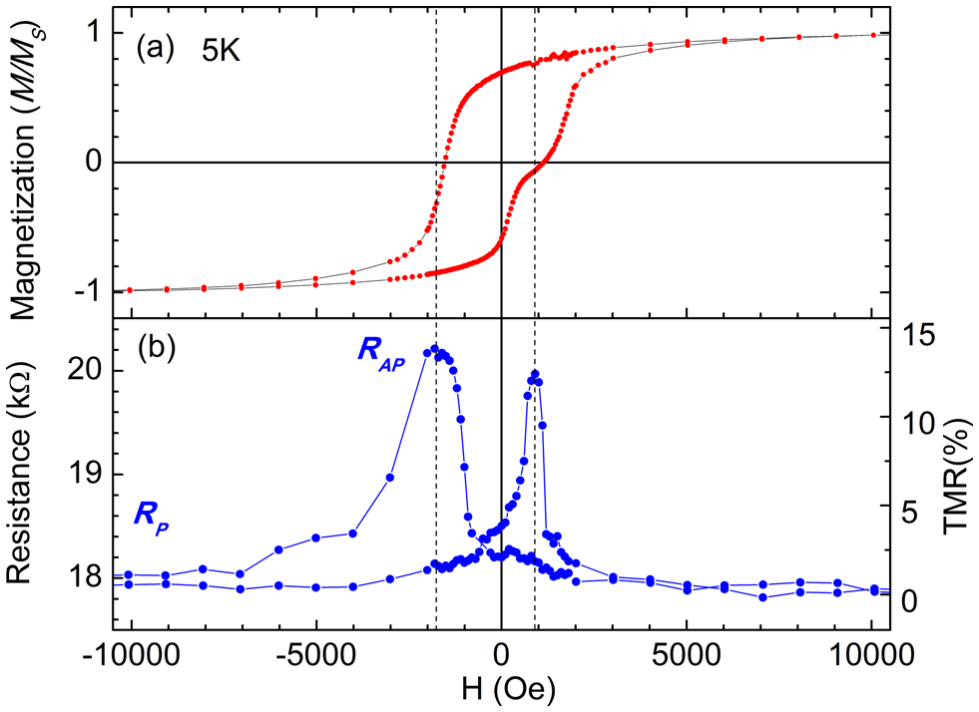
(a) The magnetic hysteresis loop of Ag(30 nm)/Co(10 nm)/CoO-ZnO(2 nm)/Co(30 nm)/Ag(60 nm) junction measured at 5 K. (b) The tunneling magnetoresistance of the junction measured at 5 K.

Figures 3a and 3b further show the magnetic hysteresis loop and the corresponding TMR loop of Ag/Co/CoO-ZnO/Co/Ag junction measured at 5 K. The exchange bias field is about 420 Oe at 5 K, which can be estimated from the shift of both loops. The TMR ratio is increased to 13.3% at the peak value. The magnetization reversals of the bottom and top Co layers are further modulated by the exchange bias field, as shown in Figure 3a. The coercivity (or magnetization reversal) of both Co layers further separates from each other at the side of positive magnetic field, but it becomes close at the side of negative magnetic field. Correspondingly, the TMR loop in Figure 3b shows sharper peak at the side of positive magnetic field, indicating higher sensitivity of TMR at this side if used as a magnetic sensor.

## Discussion

**Figure 4:**
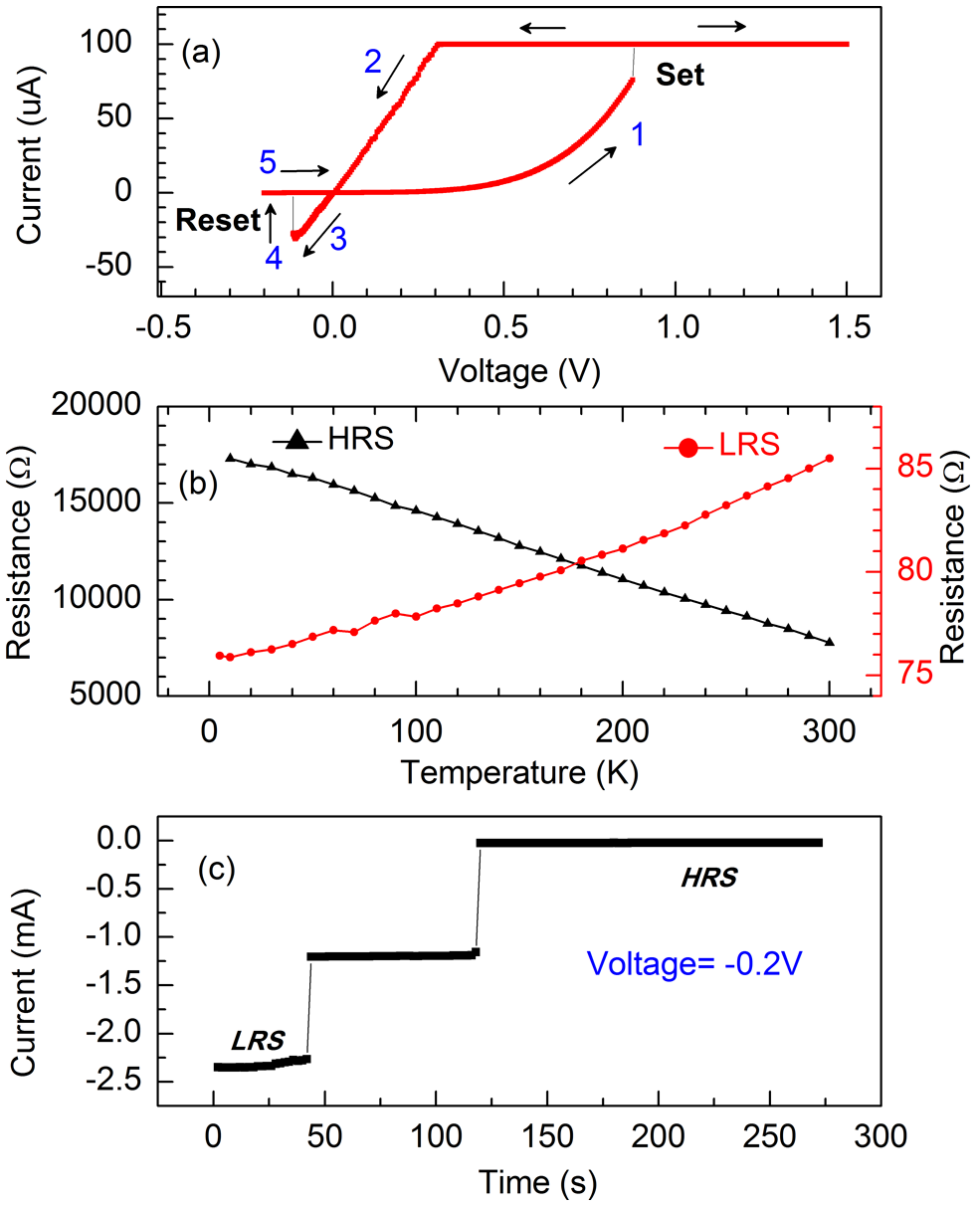
(a) The I-V characteristic of glass/Cr(2 nm)/Ag(30 nm)/Co(10 nm)/CoO-ZnO(2 nm)/Au(60 nm) junction measured at 300 K. A current limiter of 100 uA was used. (b) The temperature dependent resistance of Ag(30 nm)/Co(10 nm)/CoO-ZnO(2 nm)/Co(30 nm)/Ag(60 nm) junction, which was measured using very small currents of 0.1 uA for the resistance in HRS and 10 uA for the resistance in LRS, respectively. (c) The time dependence of electrical switching of Ag(30 nm)/Co(10 nm)/CoO-ZnO(2 nm)/Co(30 nm)/Ag(60 nm) junction measured at 300 K. All measurements were carried out without magnetic field. The magnetic orientation of the Co magnetic layers is in the remanent magnetization state during the measurements.

We also fabricated glass/Cr(2 nm)/Ag(30 nm)/Co(10 nm)/CoO-ZnO(2 nm)/Au(60 nm) reference junction in which an inactive Au electrode replaced the electrochemically active top Co/Ag bilayer. The bipolar switch of this junction is shown in Figure 4a, which is similar to that observed in Figure 2a. This means that the top ferromagnetic metal Co layer is not required for the observation of the bipolar switch, though it is necessary for tunneling magnetoresistance. Figure 2a and Figure 4a further indicate that the amplitude of the set voltage is always bigger than that of the reset voltage. This means that the HRS is more stable than the LRS. Similar resistive switching phenomena were attributed to the formation/rupture of metal filaments. Some experiments suggested that the metallic ions injected from the anode to the insulator may be responsible for the filament channel^5^,^26^,^27^. Since the Au electrode is inactive and the Ag ions at the bottom electrode cannot migrate into CoO-ZnO composite layers under the positive bias voltage, we can easily exclude the possibility of metallic ions migration in our case. Other experiments suggested that the electrical switching is due to the filament formation/rupture by a redox process^20^,^28^,^29^, which was caused by the migration of oxygen ions in the oxide. This filament scenario can also be excluded in our case based on the following analysis.

In order to elucidate the possible conduction mechanism of the Co/CoO-ZnO/Co MTJ, we examined the temperature dependence of HRS and LRS resistance. As shown in Figure 4b, the HRS resistance decreases with increasing temperature, which is a typical feature of tunneling transport through a continuous insulating barrier^30^. Correspondingly, the nonlinear I-V curve in the HRS in Figure 2a also shows tunneling transport through a continuous insulating barrier. On the contrary, the LRS resistance increases with increasing temperature, showing metallic-like transport behavior. Moreover, the linear I-V curve in the LRS in Figure 2a shows pure ohmic transport behavior. However, the LRS resistance does not increases linearly with increasing temperature, which means that the conducting paths are still not the pure metal or alloy filaments.

Figure 4c further indicates the time dependence of the electrical switching by applying a voltage less than the reset voltage in amplitude to obtain the HRS. The switching shows multiple plateaus, which are similar to the multilevel switch in TiN/ZnO/Pt^31^. The time dependence of the electrical switching shows two obvious features: first, within each plateau, the resistance (R = V/I) increases very slowly with increasing time; second, the sharp resistance jumps between plateaus indicate that the metal-insulator transition happens at least in some areas of the CoO_1−v_ layer and finally in the whole CoO_1−v_ layer.

**Figure 5:**
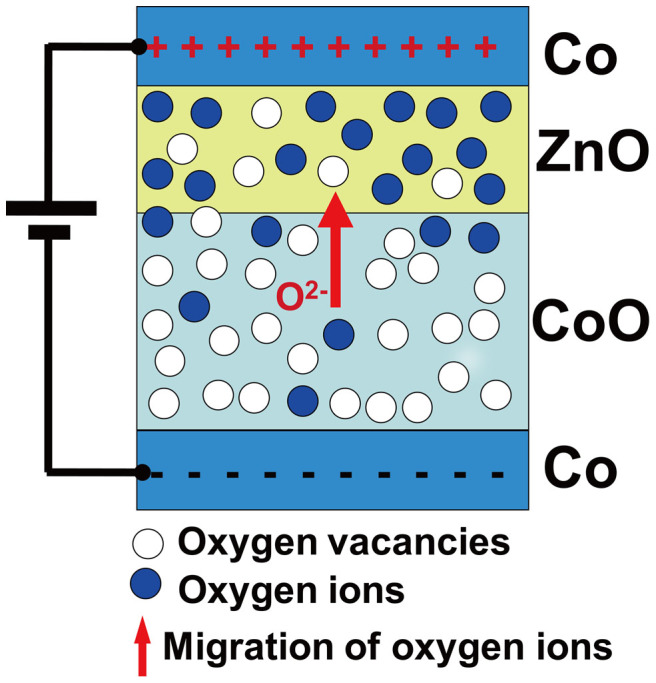
Schematics of the migration of oxygen ions between very thin CoO and ZnO layers under a positive voltage, and resulting metal-insulator transition of CoO_1−v_ in Co/CoO-ZnO/Co junctions. Due to the migration of oxygen ions from the CoO_1−v_ layer to the ZnO_1−v_ layer, the system involves from the high resistance state to the low resistance state under the positive voltage. Co and Zn atoms are not shown to simplify the schematics.

Taking into account all the above experimental results, we proposed a new scenario of migration of oxygen ions and resulting metal-insulator transition of CoO_1−v_ to explain the observed electrical switching and magnetoresistance, which is depicted schematically in Figure 5. ZnO is usually an n-type semiconductor ZnO_1−v_ and has relatively small resistivity at room temperature due to the existence of O vacancies. By contrast, CoO is usually a p-type semiconductor Co_1−x_O (0 ≤ x < 1 denotes Co vacancies) due to the existence of Co vacancies (Co-deficient or O-excess)^22^. However, in an O-deficient case like Co/CoO-ZnO/Co junctions, CoO can be in the form of CoO_1−v_ and has a trend to become more insulating by obtaining O ions. In this case, the change in junction resistance mainly depends on the CoO layer rather than ZnO layer. Assuming the initial HRS is the insulating CoO and/or O-deficient CoO_1−v_ which has much larger resistance than that of the ZnO_1−v_, the resistance is mainly due to the direct tunneling of electrons through CoO and indirect tunneling through the oxygen vacancies. Under a positive bias voltage, oxygen ions (O^2−^) leave the CoO_1−v_ layer to ZnO_1−v_ layer, and make the CoO_1−v_ layer have more oxygen vacancies. This increases indirect tunneling paths through oxygen vacancies and reduces the resistance. With increasing the concentration of oxygen vacancies, the initial localized wave functions of the electrons trapped in the oxygen vacancies overlap and become delocalized. In this case, the insulating or semiconducting CoO_1−v_ of HRS becomes metal-like CoO_1−v_ of LRS. Since this metal-insulator transition may happen in the whole CoO_1−v_, both the HRS and LRS show electrical transport properties through a whole junction area. Vice versa, a negative bias voltage can turn the metal-like CoO_1−v_ of LRS into the insulating or semiconducting CoO_1−v_ of HRS.

According to the above model, two features of Figure 4c can be well explained. First, within each plateau, the resistance increases very slowly with increasing time. This means that the migration of oxygen ions (corresponding to the migration of oxygen vacancies) is much slower at a voltage less than the reset voltage in amplitude. In fact, the current due to the migration of oxygen vacancies is negligibly small as compared with the current due to the electron movement in the metal-like LRS. The decrease of the oxygen vacancies in the metal-like CoO_1−v_ layer mainly reduces the carrier density, which leads to a slow increase of the resistance. Second, the sharp resistance jumps between plateaus indicate that the metal-insulator transition happens at least in some areas of the CoO_1−v_ layer and finally in the whole CoO_1−v_ layer. When the concentration of oxygen vacancies in the CoO_1−v_ layer is less than a critical value, the delocalized wave functions of the electrons suddenly become localized and the conducting electrons were trapped in the oxygen vacancies. In this case, the metal-like CoO_1−v_ of LRS becomes insulating or semiconducting CoO_1−v_ of HRS.

Finally, we briefly discussed the potential applications of the spin memristive Co/CoO-ZnO/Co MTJ devices. The spin memristive MTJ devices may have applications in multibit nonvolatile data storage and artificial neuronal computing. It is well known that MTJ is the basic storage element of magnetic random access memory with high-speed and nonvolatile memory, and meanwhile resistance switching is utilized to develop resistive random access memory. By both electrical and magnetic controlling, the multiple resistance states of the spin memristive MTJ devices can greatly increase the data storage densities. Another exciting application is that the spin memristive MTJ may work as nanoscopic synapse-neuron system. A detailed discussion about the memory effects in complex materials and nanoscale systems can be found in the recent review article^32^.

In conclusions, we have successfully fabricated spin memristive Co/CoO-ZnO/Co junctions with CoO-ZnO nanon composite barrier layers, which simultaneously realize large memristance and tunneling magnetoresistance. The bipolar resistance switching ratio is high up to 90, and the TMR ratio of the high resistance state gets to 8% at room temperature. The bipolar resistance switching is explained by the migration of oxygen ions and resulting metal-insulator transition of CoO_1−v_ layer. The spin memristive devices have promising applications in multibit nonvolatile data storage and artificial neuronal computing.

## Methods

The Co/CoO-ZnO/Co MTJs with an area of 0.1 mm × 0.1 mm were deposited by a magnetron sputtering machine with a base pressure of 6 × 10^−8^ Torr using shadow masks. The bottom Co layer of 10 nm was deposited under an Ar gas of 5 × 10^−3^ Torr. Then a semiconducting ZnO layer of 2 nm was deposited by RF reactive sputtering of ZnO target under the argon-oxygen mixture of 6 × 10^−3^ Torr with 0.3% oxygen ratio. An antiferromagnetic insulating CoO thin layer was produced on the surface of the bottom Co layer during the deposition of ZnO. Finally the top 30 nm Co layer with 60 nm Ag electrode was grown at a high Ar pressure of 1.5 × 10^−2^ Torr, resulting in a larger coercivity (H_C_ = 310 Oe) at room temperature than that of the bottom Co layer (H_C_ = 70 Oe) grown at lower pressure.

XPS was performed on a Thermo Scientific ESCALAB 250XI photoelectron spectrometer. The spectra were calibrated by using the adventitious carbon C1s (284.6 eV) peak. The etching rate by 1000 eV argon ions is 0.08 nm/sec. The XPS measurements of Co, O, Zn, and Ag elements were carried out in a vacuum chamber when the glass/Cr(2 nm)/Ag(30 nm)/Co(10 nm)/CoO-ZnO(2 nm) film was etched by Ar^+^ gradually from the ZnO layer through CoO to Co layer. The atomic percent of Co, O, Zn, and Ag elements in different layers was measured by XPS, and the chemical valence of Co element in Co and CoO layers was confirmed. The magnetic properties were characterized with a SQUID magnetometer with magnetic field high up to 7 T parallel to the substrate. The samples were cooled down to 2 K from 300 K without magnetic field.

The electrical transport measurements were carried out in a four-point configuration in a Quantum Design instrument with the Model 2400 source meter instrument and Model 2182A nano voltmeter. The bottom and top electrode contact resistance is negligible as compared with the junction resistance, which eliminates the current crowding.

## Author Contributions

S.S.Y., L.M.M. and P.G. proposed the concept. Q.L. designed and conducted the experiments with T.T.S., Y.L.C., K.Z. and Y.F.T. X.L.W., S.S.K., M.W.Z., Y.X.C., G.L.L. and Y.Y.D. contributed to the analysis and discussion for the results. S.S.Y. and Q.L. wrote the paper and all the co-authors comment on it.
